# Title Current Status of the Search for Biomarkers for Optimal Therapeutic Drug Selection for Patients with Rheumatoid Arthritis

**DOI:** 10.3390/ijms22179534

**Published:** 2021-09-02

**Authors:** Haruka Tsuchiya, Keishi Fujio

**Affiliations:** Department of Allergy and Rheumatology, Graduate School of Medicine, The University of Tokyo, Tokyo 113-0033, Japan; fujiok-int@h.u-tokyo.ac.jp

**Keywords:** rheumatoid arthritis, biomarkers, precision medicine, synovial biopsy

## Abstract

Rheumatoid arthritis (RA) is an autoimmune disease characterized by destructive synovitis. It is significantly associated with disability, impaired quality of life, and premature mortality. Recently, the development of biological agents (including tumor necrosis factor-α and interleukin-6 receptor inhibitors) and Janus kinase inhibitors have advanced the treatment of RA; however, it is still difficult to predict which drug will be effective for each patient. To break away from the current therapeutic approaches that could be described as a “lottery,” there is an urgent need to establish biomarkers that stratify patients in terms of expected therapeutic responsiveness. This review deals with recent progress from multi-faceted analyses of the synovial tissue in RA, which is now bringing new insights into diverse features at both the cellular and molecular levels and their potential links with particular clinical phenotypes.

## 1. Introduction

Rheumatoid arthritis (RA) is an autoimmune disease that can impair physical function by causing persistent synovial inflammation, leading to joint destruction. In RA pathogenesis, various molecules in immune cells (e.g., T-cells, B-cells, and monocytes) and mesenchymal cells are dysregulated through the influence of genetic predisposition and environmental factors. From the early onset of RA to the progression of destructive synovitis, these pathogenic cells cooperate and activate each other directly via cell-to-cell contact, or indirectly via humoral factors (e.g., cytokines and chemokines). For instance, tumor necrosis factor (TNF)-α, interleukin (IL)-6, and IL-1β play major roles in joint inflammation. In response, the field of RA therapy has developed disease-modifying antirheumatic drugs (DMARDs) that can be divided into biological DMARDs (bDMARDs) and targeted synthetic DMARDs (tsDMARDs) [1]. The former class includes TNF-α inhibitors, the T-cell costimulation inhibitor (CTLA4Ig; abatacept), the IL-6 receptor inhibitors, and the antibody targeted against CD20 (rituximab). The latter includes Janus kinase inhibitors. Randomized controlled trials (RCTs) have proven the effectiveness of each of these drugs and, certainly, it is no exaggeration to say that these options have revolutionized the daily practice of RA treatment. However, approximately 40% of RA patients are still treated ineffectively with the existing DMARDs. In patients who previously had an inadequate response to conventional synthetic DMARDs (csDMARDs), typically methotrexate, the outcome measures (American College of Rheumatology 20% response rate (ACR20), ACR50, and ACR70) of bDMARDs or tsDMARDs have only been reported in about 60%, 40%, and about 20% of patients, respectively [2].

RA patients are diverse, varying by symptom severity, affected joints, and autoantibodies. Heterogeneity in treatment responsiveness is also common and poses clinical challenges. Current treatment strategies are exploratory, and clinicians continue to approach treatment empirically by administering various combinations of drugs until they find an effective drug. This inefficient approach takes time, and during this time, there can be irreversible joint destruction. Therefore, the search for biomarkers that predict the treatment responsiveness of individual patients is an urgent task, and success here should accelerate the next generation of RA therapy by optimizing the allocation of therapeutics. In this review, we discuss the recent progress that provides a foundation for precision medicine for RA patients.

## 2. The Search for Therapeutic Response Predictors Using Information Obtained from Peripheral Blood and Minimally Invasive Tests

### 2.1. Acute Phase Reactants

To date, peripheral blood has been used in many translational studies to identify treatment-responsive biomarkers because of the ease of its collection, which is less invasive and easily repeatable. For serum protein components, a recent pilot study of 298 patients with early RA (the Swedish Pharmacotherapy (SWEFOT) clinical trial) identified that low baseline levels of C-reactive protein (CRP) and leptin, and high baseline levels of tumor necrosis factor receptor I (TNF-RI) and vascular cell adhesion molecule 1 (VCAM-1), potentially predicted the response to methotrexate, that is, disease activity, based on the Disease Activity Score in the 28-joint erythrocyte sedimentation rate (DAS28-ESR) < 3.2, at a 3-month follow-up examination [3]. Focusing on TNF-α inhibitors, a 52-week, randomized, double-blind phase IIIb study of 194 RA patients with an inadequate response to csDMARDs demonstrated that higher levels of pre-treatment inflammatory markers (CRP) were associated with a better treatment response for a TNF-α inhibitor [4]. In contrast, there was no significant association between inflammatory markers at the baseline (CRP and ESR) and treatment response to IL-6 receptor inhibitors. In post hoc analyses of three RCTs [5,6,7] and a pooled analysis of five RCTs including 4186 RA patients, no association was found between levels of inflammatory markers before the administration of a therapeutic drug and response to IL-6 receptor inhibitors. One prospective, observational study focused on the association between levels of inflammatory markers and IL-6 receptor inhibitor retention, reporting that patients with higher levels of CRP at baseline were linked to lower discontinuation rates [8].

### 2.2. Autoantibodies

The presence of autoantibodies (i.e., rheumatoid factor (RF) and anti-cyclic citrullinated peptide antibody (anti-CCP)) predicts the response to anti-CD20 antibody (rituximab) [9] and CTLA4-Ig (abatacept) [10,11]. Specifically, a meta-analysis of four placebo-controlled, phase II or III clinical trials (rituximab, *n* = 1416; placebo, *n* = 761) indicated that seropositive patients respond better to rituximab than seronegative patients [9]. Moreover, in an observational study of 566 patients with RA who received abatacept, and 1715 who received TNF-α inhibitors, anti-CCP-positive abatacept initiators were associated with significantly better treatment response than anti-CCP-negative abatacept initiators, but no significant difference was observed for TNF-α inhibitor initiators [11]. However, the value of autoantibodies as factors predicting the therapeutic responsiveness of TNF-α inhibitors and IL-6 receptor inhibitors has not been established due to variability in the results [12,13].

### 2.3. Genome, Epigenome, and Gene Expression Signatures

From a genomic viewpoint, recent genome-wide association studies (GWAS) have identified more than 100 susceptibility loci in RA [14]. Although the causal relationship between these “risk single nucleotide polymorphisms (SNPs)”, genomic coordinates, and the final trait “disease” is robust, the whole picture of the relationship between genomic variation and treatment responsiveness has not been revealed. Several studies have reported gene polymorphisms involved in treatment responsiveness to specific drugs (e.g., steroids [15], methotrexate [16,17] TNF-α inhibitors [18,19], and IL-6 receptor inhibitors [20,21,22,23]), and these findings are awaiting further verification.

Therapeutic changes in epigenome modification (e.g., DNA methylation) and their usefulness as biomarkers have been reported in a few studies. From the perspective of DNA methylation in whole blood, the changes observed after 4 weeks of methotrexate treatment in four CpG sites (Adrenoceptor Alpha 2C (*ADRA2C*), GATA Binding Protein 3 *(GATA3*), MicroRNA 181 (*MIR181*), small nucleolar RNA, C/D Box 84 (*SNORD84*)) were associated with therapeutic response at 6 months [24]. In addition, by utilizing GWAS summary statistics, heritable immune cell traits, whole blood gene expression, and DNA methylation, a new methodological approach for localizing genetic effects for a response to TNF inhibitors was promoted [25].

Other studies have suggested that the expression of type I interferon (IFN)-signaling-related genes is upregulated in the peripheral blood and synovium of certain RA populations [26]. Raterman et al. reported that the high expression of type I IFN network genes in the peripheral blood at baseline was associated with treatment resistance to rituximab [27], whereas Wampler et al. found that higher IFN signaling in neutrophils correlates with a good response to TNF-α inhibitors [28]. Recently, Wampler et al. monitored serum type I IFN activity, focusing on IFN-α and IFN-β activity using a functional reporter cell assay in RA patients just prior to them being treated with a TNF-α inhibitor [29]. Higher IFN-β activity was observed in the TNF-α inhibitor non-responsive group, and an increased ratio of IFN-β to IFN-α (IFN-β/α activity ratio) in the pretreatment serum was associated with a lack of response to TNF inhibition. Interestingly, no patient with a ratio of ≥ 1.3 achieved clinical remission or low disease activity (77% specificity and 45% sensitivity for the prediction of non-response).

### 2.4. Obesity

High Body Mass Index (BMI) is common (> 60%) among patients with RA [30], and it is associated with higher disease activity and disability [31,32]. The impact of obesity on the effectiveness of bDMARDs appears to vary between drug classes [33].

In a prospective study, Klaasen et al. assessed the impact of baseline BMI on the clinical response to TNF-α inhibitors in 89 active RA patients [34]. The BMI correlated positively with the DAS28 at the baseline and a significant, negative association between the BMI and the absolute decrease in the DAS28 after 16 weeks of treatment was found. In fact, only about half of obese patients achieved good response (84% and 50% for normal and obese patients, respectively). In an Italian multicenter registry that included 641 patients treated with TNF-α inhibitors [35], a lower percentage of obese patients achieved clinical remission after 12 months (32.9% and 15.2% for normal and obese patients, respectively). Consistent with these results, obesity lowered the chance of attaining clinical remission in post hoc analyses of RCTs [36,37].

Previous studies on the IL-6 receptor inhibitors abatacept and rituximab did not confirm a clear association between therapeutic responses with BMI compared to TNF-α inhibitors. In a retrospective study including 200 RA patients [38], there was no significant association between the response to IL-6 receptor inhibitors at 6 months, and the baseline BMI. Another retrospective, multicenter study reported similar results. Specifically, in a study of 115 patients, the median baseline BMI did not differ between responders and non-responders after 6 months of treatment [39]. Moreover, in a post hoc analysis of a 6-month-long prospective study (ACTION trial), the influence of baseline BMI on the efficacy and retention rate of abatacept was investigated in 643 bDMARD-treated, treatment-naïve patients [40]. At baseline, the obese group had more active disease; the mean DAS28-CRP was 4.6 and 5.1 for the underweight/normal and obese groups, respectively. There were no significant associations in the proportion of responders (80.7% and 77.0% for the underweight/normal and obese groups, respectively) or overall retention rates (89% for the underweight/normal and obese groups) at 6 months based on BMI stratification. Similar results were reproduced in a post hoc analysis of a 6-month-long prospective study (ACQUIRE trial) involving 1456 patients treated with abatacept [41], and there were no significant differences in clinical remission rates at 6 months across BMI groups. Furthermore, in a pooled analysis of 10 prospective cohorts involving 2015 patients treated with abatacept, the median retention time (1.91 years for obese groups compared to 2.12 years for non-obese groups) and drug discontinuation risk (HR 1.08 (95% CI 0.89–1.30) for obese patients compared to normal-weight patients) was not associated with BMI [42]. Obese and non-obese patients attained similar rates of clinical response to abatacept at 6 or 12 months. Finally, a retrospective study evaluated the effectiveness of rituximab according to body weight in 114 RA patients [43]. The median baseline BMI was comparable among responders and non-responders at 6 months, and the clinical response was not different across categories of BMI after 6 months (21.1% for the obese group and 23.7% for the non-obese group).

## 3. Approach to Information Obtained from Local Joints

So far, biomarkers for the examination of therapeutic responsiveness have been searched for using synovial fluid (SF), which is in close proximity to synovial tissue and has significant potential to help better understand underlying disease pathogeneses. Helen et al. measured 12 cytokine concentrations in the SF of patients with inflammatory arthritis (42 RA patients and 19 non-RA patients) and they reported that the SF from RA patients contained significantly elevated levels of a wide variety of cytokines (e.g., IL-1β, IL-17, IFN-γ and TNF-α) compared with the SF from non-RA patients [44]. Moreover, RA patients who did not respond to TNF-α inhibitors had elevated IL-6 in their SF at baseline, whereas responders had elevated IL-2 and G-CSF. Furthermore, the recent technological developments in SF proteomics drive the search for biomarkers, and the identification of post-translational modifications and targeted proteins can stratify patients for therapy selection [45]. Several mass spectrometry-based SF proteomics studies revealed increased levels of some proteins (e.g., 14-3-3 zeta/delta and 14-3-3 eta) in the SF from RA patients in comparison to osteoarthritis patients [46,47,48,49]. Hilde et al. reported that the concentration of calprotectin (heterocomplex of S100A8/A9 proteins) in SF showed significant association with RA disease activity assessed by ultrasound or clinical examination. In addition, decreased calprotectin at the first month after the initiation of bDMARDs was predictive of therapeutic responsiveness at 3, 6 and 12 months [50].

## 4. Usefulness and Safety of Ultrasound-Guided Synovial Needle Biopsy

In parallel with the progress in research using peripheral blood, there has been a growing recognition of the need for information obtained from the inflamed synovium, the predominant target tissue in RA [51,52,53]. Recently, synovial biopsies for RA patients have been performed not only for the purpose of diagnosis (e.g., to exclude infectious arthritis or crystal arthritis), but also for research purposes (e.g., to elucidate the pathogenetic mechanisms, evaluate response to therapy, and aid the search for novel drug discovery targets). There are three major established methods for synovial biopsy: blind needle biopsy, arthroscopy-directed biopsy, and ultrasound-guided needle biopsy [54]. Blind needle biopsy has been used to obtain synovium for several decades, and the safety and feasibility of the procedure is well established [55]. The advantages of this technique are its cost effectiveness and the fact that it does not require special equipment. However, there are also some disadvantages: (1) it is not possible to directly visualize the tissue to be biopsied; (2) the joint from which synovium can be collected by this procedure is usually limited to the knee joint, especially from the suprapatellar bursa; (3) synovial sampling might fail, especially in joints where inflammation has subsided. In contrast, since arthroscopy-directed synovial biopsy allows direct viewing of the synovium, it rarely fails to collect synovial membrane, even in clinically quiescent joints. However, its disadvantages are that it is more expensive than the needle biopsy technique and can only be performed in a facility equipped with technicians and a specialized sterile area.

More recently, minimally invasive ultrasound-guided needle biopsy has become widely accepted. This approach permits access to the synovial tissue in various joints, including small and medium-sized joints, no advanced equipment is required, and the procedure is relatively inexpensive compared to arthroscopy. In a European multicenter study, comparisons were made between the safety profiles and patient-reported outcomes (PRO) of patients undergoing ultrasound-guided needle biopsy, blind needle biopsy, or arthroscopy-directed biopsy. A total of 524 synovial biopsies were performed, and there were no significant differences between different methods in the frequency of adverse events or changes in PRO after biopsy [56]. Another study evaluating 64 patients who underwent ultrasound-guided needle biopsy also reproduced the safety and tolerability of the procedure, and synovial tissue was retrieved in 88% of biopsies, with a median of 75% gradable samples [57].

## 5. Synovial Information That Directly Reflects Local Inflammation

With the establishment of safe protocols and the widespread use of ultrasound-guided synovial needle biopsy procedures, it is becoming possible to understand the pathophysiology of synovitis in real time, sometimes in chronological order (e.g., before and after treatment). In particular, information on synovial tissue in clinically well-characterized populations provides hints for selecting effective therapeutic agents for individual patients.

To date, the histopathological heterogeneity of synovial tissue (e.g., lining layer hypertrophy, angiogenesis, and immune cell infiltration) in RA has been reported. For instance, Humby et al. pathologically classified the synovium of early-stage, treatment-naïve RA patients into three types: lympho-myeloid (dominated by the presence of B-cells in addition to myeloid cells), diffuse-myeloid (with myeloid lineage predominance but poor in B-cells), and pauci-immune (characterized by scanty immune cells and prevalent stromal cells) [58]. Lymphoid aggregates express activation-induced cytidine deaminase (a DNA-editing enzyme necessary for somatic hypermutation and class-switch recombination of immunoglobulin genes in B-cells) and are surrounded by anti-CCP producing plasma cells, indicating the local production of autoantibodies [59,60].

Technological advances have enabled more precise analysis, and efforts are being made to link synovial histopathology with infiltrating cell types, molecular pathways, and clinical phenotypes, including therapeutic responsiveness [58,61,62,63]. For instance, compared with (*n* = 78) and without (*n* = 45) anti-CCP in serum, synovium from anti-CCP-positive RA patients was characterized by higher numbers of lymphoid aggregates of CD19^+^ B-cells. The CD68^+^ macrophage and CD8^+^ T-cell infiltrate levels were predictive of a good response to TNF-α inhibitors [61]. In another study, 144 early-stage, treatment-naïve RA patients underwent synovial biopsy before, and 6 months after, the initiation of DMARDs, and the histopathological information (i.e., lympho-myeloid, diffuse-myeloid, and pauci-immune) and gene expression profiles were analyzed in an integrated manner [58]. An elevation of myeloid- and lymphoid-associated gene expression scores strongly correlated with disease activity, acute phase reactants, and DMARD response at 6 months. Furthermore, the elevation of osteoclast-targeting genes predicted radiographic joint damage progression at 12 months. Patients with pauci-immune histopathology showed less severe disease activity and joint destruction. The combination of histopathological findings and gene expression information also improved the prediction of biological therapy requirements at a 12-month follow up [63]. In a study involving 37 patients with established RA who used TNF-α inhibitors, patients with a myeloid pattern of pretreatment synovial pathology were more responsive to certolizumab than patients with a pauci-immune pattern [64]. Dennis et al. categorized RA synovium into four major phenotypes according to gene expression patterns: lymphoid, myeloid, low inflammatory, and fibroid [65]. Importantly, higher myeloid but not lymphoid scores at baseline predicted good clinical response to TNF-α inhibitors at 6-week follow up. They also reported that patients with high baseline serum soluble intercellular adhesion molecule 1 (sICAM1)/low C-X-C motif chemokine 13 (CXCL13) had a 42% probability of an ACR50 response to TNF-α inhibitors vs. 13% in those with low sICAM1/high CXCL13.

Focusing on B-cell infiltration of the synovium, Rivellese et al. compared the synovial biopsies of 165 early-stage, treatment-naïve RA patients with those of 164 established RA patients with an inadequate response to TNF-α inhibitors (TNFi-IR) [66]. B-cells abundantly infiltrated the synovium of TNFi-IR patients compared with treatment-naïve patients, and significantly higher histopathological synovitis scores in B-cell-rich patients were observed in both groups. Building on these findings, attempts to predict the therapeutic responsiveness of rituximab and tocilizumab in TNFi-IR patients have been conducted in a more precisely controlled strategy (R4-RA): a 48-week, biopsy-driven, phase 4, open-label, multicenter, randomized controlled trial [67]. A total of 164 patients (128 (78%) female; median age 55.5 years (interquartile range; IQR 47.4 to 65.3)) with RA underwent a synovial biopsy before therapeutic intervention and were classified as B-cell poor or B-cell rich based on histological findings. Subsequently, patients were randomly assigned to the rituximab (83 (51%) patients) or the tocilizumab (81 (49%) patients) group. Baseline synovial samples were also subjected to bulk RNA sequencing and reclassified using the B-cell molecular signature. Following histological ‘B-cell poor’ classification, the Clinical Disease Activity Index (CDAI) 50% response rate was not significantly different between the rituximab group (17 (45%) of 38 patients) and tocilizumab group (23 (56%) of 41 patients; *p* = 0.31). However, following reclassification using the expression level of B-cell related molecules (as measured by bulk RNA sequencing), the CDAI 50% response rate was significantly higher in the tocilizumab group compared with the rituximab group (rituximab group: 12 (36%) of 33 patients vs. tocilizumab group: 20 (63%) of 32 patients; *p* = 0.035). No significant difference in adverse events was observed between the therapeutic agents. These results suggest that gene expression-based stratification of the RA synovium could be useful in predicting treatment responsiveness and drug selection in a group with a uniform baseline. Unfortunately, however, the trial was not statistically powered to show the advantage of rituximab in the B-cell-rich population, which means that there was still a problem in estimating the number of rituximab responders.

From another point of view, recent progress in mass cytometry of CD4^+^ T-cells isolated from RA patients’ synovia discovered a hitherto unidentified population of PD-1^hi^CXCR5^-^, described as ‘peripheral helper’ T-cells (TPH) that promote B-cell responses and induce plasma cell differentiation in vitro [68]. Improvements in disease activity were correlated with a decrease in this pathogenic fraction. Zhang et al. reported that TPH cells were overabundant in inflamed (so-called leukocyte-rich) RA synovia compared with the synovia of OA and healthy controls [69]. In a study of 11 RA patients starting TNF-α inhibitors, synovial biopsies were performed at baseline and at a 20-week follow up. Based on the transcriptomic analysis with cell type deconvolution, a lower abundance of PD-1^hi^CXCR5^-^ peripheral helper T-cells in the synovium was associated with a good response to TNF-α inhibitors [70].

## 6. Precise Analysis of Synovium by Technological Development

Recent developments in single-cell RNA sequencing technology have dramatically improved our understanding of the RA synovium. Among these analyses, some of the most detailed studies have been of synovial fibroblasts (SFs), which are multifunctional mesenchymal cells in the joint. In the normal joint, SFs produce substrate proteins (e.g., fibronectin, collagen) and extracellular matrix degrading enzymes (e.g., proteases) to maintain the synovial structure. SFs also contribute to synovial fluid composition by producing joint lubricants (e.g., hyaluronic acid), and provide nourishment to the underlying articular cartilage. Meanwhile, in the inflamed synovium of RA, SFs that have acquired activated phenotypes triggered by cell-to-cell interactions and humoral factors invade the adjacent articular cartilage. SFs highly express various adhesion molecules and proinflammatory and matrix-degrading mediators, and among them, they are known as a major source of IL-6 in joints [69]. Moreover, SFs stimulate angiogenesis in the synovium through the production of proangiogenic factors, which promote the infiltration of immune cells and contribute to the persistence of joint inflammation.

It has long been debated whether SFs are a uniform cell population. Mizoguchi et al. reported that single-cell RNA sequencing of RA synovia classified synovial SFs into at least three subpopulations [71]. It was suggested that CD34^-^THY1^+^ SFs (localized around the blood vessels in the sub-lining) are a pathological subpopulation that produce inflammatory cytokines (e.g., *IL6*). Following this study, the Accelerating Medicines Partnership (AMP), an active partner of the U.S.’ National Institutes of Health (NIH) since 2014, utilized an integrated approach of single-cell RNA sequencing, mass cytometry, bulk RNA sequencing, and flow cytometry of dissociated RA synovia, and reported that CD34^-^THY1^+^HLA-DR^high^ SFs are a pathological subpopulation that highly expresses IL-6 [69]. In addition, a study on a mouse model suggested that FAPα^+^THY1^+^ SFs localize to the sub-lining and are involved in synovial inflammation, while FAPα^+^THY1^-^ SFs in the lining are involved in bone destruction [72]. Based on these findings, Wei et al. reported that lining and sub-lining fibroblasts exist along a gradient that corresponds to the anatomical localization of SFs in the synovium, regulated by endothelium-derived Notch3 signaling [73]. The distribution of synovial cell types defined by this unbiased approach could provide new insights into biological differences between patients. Unfortunately, however, it has not yet been put into practical use, even with these most well-characterized mesenchymal cells.

Furthermore, multi-omics analyses, including genomic data and synovial information, have also been attempted. By integrating the microarray data of synovial biopsies from active RA patients starting TNF-α inhibitors, clinical data, and GWAS data of responsiveness to TNF-α inhibition, Aterido et al. found some coexpressed genes to be significantly associated with clinical response to TNF-α inhibition [74]. These modules were found to be significantly enriched in gene sets involved in nucleotide metabolism containing epigenetic markers from immune cells, including CD4^+^ regulatory T-cells.

## 7. Conclusions

Growing interest in synovial tissue pathophysiology, as the primary target of RA, has led to extraordinary insights into the diversity of synovial phenotypes and their association with clinical subtypes. Although the field is far from achieving the goal of the practical application of precision medicine to RA, in concordance with the increasing availability of high-throughput molecular and spatial technologies and immune profiling of individual cells within the synovium, the identification of biomarkers of treatment response is steadily progressing. By combining peripheral blood and synovial information, a more personalized approach for individual patients could be feasible, and such an approach would undoubtedly lead to improved patient outcomes.
